# Novel gene variants in Polish patients with Leber congenital amaurosis (LCA)

**DOI:** 10.1186/s13023-020-01634-y

**Published:** 2020-12-11

**Authors:** Anna Skorczyk-Werner, Zuzanna Niedziela, Marcin Stopa, Maciej Robert Krawczyński

**Affiliations:** 1 Department of Medical Genetics, Poznan University of Medical Sciences, 8, Rokietnicka St, 60-806 Poznan, Poland; 2 Department of Ophthalmology, Chair of Ophthalmology and Optometry, Poznan University of Medical Sciences, Poznan, Poland; 3 Centers for Medical Genetics GENESIS, Poznan, Poland

**Keywords:** Leber congenital amaurosis (LCA), Novel variants, SNP-microarray for LCA genes, Targeted NGS panel for LCA genes

## Abstract

**Background:**

Leber congenital amaurosis (LCA) is a rare retinal disease that is the most frequent cause of congenital blindness in children and the most severe form of inherited retinal dystrophies. To date, 25 genes have been implicated in the pathogenesis of LCA. As gene therapy is becoming available, the identification of potential treatment candidates is crucial. The aim of the study was to report the molecular basis of Leber congenital amaurosis in 22 Polish families.

**Methods:**

Single Nucleotide Polymorphism-microarray for LCA genes or Next Generation Sequencing diagnostic panel for LCA genes (or both tests) were performed to identify potentially pathogenic variants. Bidirectional Sanger sequencing was carried out for validation and segregation analysis of the variants identified within the families.

**Results:**

The molecular background was established in 22 families. From a total of 24 identified variants, 23 were predicted to affect protein-coding or splicing, including 10 novel variants. The variants were identified in 7 genes: *CEP290, GUCY2D, RPE65, NMNAT1, CRB1, RPGRIP1,* and *CRX*. More than one-third of the patients, with clinical LCA diagnosis confirmed by the results of molecular analysis, appeared to be affected with a severe form of the disease: LCA10 caused by the *CEP290* gene variants. Intronic mutation c.2991+1655A>G in the *CEP290* gene was the most frequent variant identified in the studied group.

**Conclusions:**

This study provides the first molecular genetic characteristics of patients with Leber congenital amaurosis from the previously unexplored Polish population. Our study expands the mutational spectrum as we report 10 novel variants identified in LCA genes. The fact that the most frequent causes of the disease in the studied group of Polish patients are mutations in one out of three genes that are currently the targets for gene therapy (*CEP290*, *GUCY2D,* and *RPE65*) strongly emphasizes the importance of the molecular background analyses of LCA in Polish patients.

## Background

Leber congenital amaurosis (LCA) is a rare retinal disease that is the most frequent cause of inherited blindness in children. LCA is the most severe form of all inherited retinal dystrophies (IRD) and accounts for about 5% of all IRDs. The disease typically becomes evident in the first year of life, and it is estimated that about 20% of children with visual impairment in specialized schools are affected by LCA [1]. The prevalence of the disorder is estimated to be 1 in 30,000 [2], and there can be about 1000–1200 patients suffering from LCA in Poland. To date, the following 25 genes have been implicated in the pathogenesis of LCA: *AIPL1, CABP4, CCT2, CEP290, CLUAP1, CRB1, CRX, DTHD1, GDF6, GUCY2D, IFT140, IMPDH1, IQCB1, KCNJ13, LCA5, LRAT, NMNAT1, OTX2, PRPH2, RD3, RDH12, RPE65, RPGRIP1, SPATA7, TULP1* [3]. LCA is most often inherited in an autosomal recessive manner, but in rare cases, the disease may also be transmitted as an autosomal dominant trait. The symptoms usually include severe and early visual impairment, Franceschetti's oculo-digital sign (comprising eye-poking, pressing, and rubbing), nystagmus, and sluggish or near-absent pupillary responses. Other typical ophthalmological features associated with LCA are photophobia, refraction defects, nyctalopia, keratoconus, and cataract. The electroretinogram (ERG) is characteristically "nondetectable" or severely subnormal [2]. LCA's clinical symptoms are very often similar to these of different retinal dystrophies, so the accurate clinical diagnosis, especially in infants, sometimes cannot be made at the first visit or has to be revised once the molecular analysis is performed. Therefore, genetic-molecular testing is necessary to obtain a definitive diagnosis of LCA through pathogenic variant identification. Unraveling the molecular background of the disease contributes to the identification of the potential treatment gene candidates and the development of therapeutic approaches. The aim of the study was to report the molecular basis of Leber congenital amaurosis in 22 families living in Poland.

## Material and methods

### Patients, ethic statements

51 Polish families with a clinical diagnosis of LCA were referred to our genetic clinic in 2010–2019, and 44 of them had the molecular analyses. Some of these patients did not decide to order the genetic tests as they are quite expensive and not funded by the National Health Fund in Poland. Moreover, until 2016 only LCA SNP microarray based on the Arrayed Primer Extension (APEX) approach analysis (Asper Ophthalmics, Asper Biotech Ltd., Tartu, Estonia) was performed that did not allow to identify novel variants. Among patients who had LCA SNP microarray test only (35 patients), there is a group without any mutation found (16 patients), with one heterozygous variant identified in autosomal recessive LCA genes (6 patients) and the group of patients with the molecularly confirmed LCA diagnosis (13 patients). In this study, we focused on a group of 22 families (28 patients) with LCA diagnosis fully confirmed by the results of molecular analysis based on SNP microarray and NGS-LCA panel. Patients were numbered with Patient ID, where the first digit indicated the Family number and the next digit after the hyphen was the individual's laboratory number. This study was conducted in accordance with the tenets of the Declaration of Helsinki and the Association for Research in Vision and Ophthalmology (ARVO) statement on human subjects. It was also approved by the Poznan University of Medical Sciences Institutional Review Board. Written informed consent was obtained from all participants or their legal guardians.

### Clinical diagnosis

A total of 28 patients from 22 unrelated families living in Poland (21 families of Polish ethnicity and 1 of Romany origin) with a clinical diagnosis of LCA confirmed by molecular analysis results were evaluated in this study. Ophthalmologic examinations, including best-corrected visual acuity (BCVA) and funduscopy, were performed in all the probands. Electroretinography (ERG) was performed in 24 patients (it was not performed in patients: 4–11, 11–36, 15–44, 17–57). Spectral-Domain Optical Coherent Tomography (SD-OCT) retinal scans (Optovue, Fremont, CA, USA) were obtained in four families: Family no. 6 (patients 6–18, 6–19, 6–52), Family 10 ( 10–33), Family 13 (patients 13–53 and 13–54) and Family 17 (patient no.17–57). Fluorescein angiography (FA) was done in three patients: 10–33, 13–53, and 17–57. In four patients, we conducted perimetry: 10–33, 13–53, 17–57, and 22–50. Magnetic resonance imaging (MRI) of the head and eye orbits were obtained during early infancy in seven subjects: 3–7, 5–15, 8–25, 9–29, 12–39, 16–56, 20–59. Some patients, especially older ones, do not remember whether they underwent MRI. The symptoms observed in our study group are listed in Table 1.

**Table 1:** Clinical symptoms and the results of the ophthalmological examinations in 22 Polish families with LCA

Patient ID	Current age (years)/gender	Disease onset and first symptoms	BCVA OD OS	Ophthalmological symptoms	Fundus appearance (age at the funduscopy or other examination)	ERG results	Other non-ocular symptoms
1–1	38/F	1 year—nystagmus	1/50 Light perception	Photophobia, high hyperopia, keratoconus in both eyes	Retinal dysplasia with attenuated retinal vessels and optic nerves atrophy (29 years)	Bilaterally flat ERG tracings	–
2–4	3/F	1 month—nystagmus the oculo-digital sign	Light perception	High hyperopia (+ 9.0 D) convergent strabismus, no fixation, minimal eye contact	Pale optic nerve disc, salt and pepper appearance of the eye fundus, attenuated vessels, loss of macular reflex (3 years)	Photopic diminished, scotopic extinguished	Muscular hypotonia after birth
3–7	8/M	2 months—nystagmus, the oculo-digital sign, no fixation, no pacing	Light perception (intense light only)	Deep-set eyeballs, nystagmus, mild photophobia, hyperopia (+ 4.0 D)	Normal optic nerve head, retinal pigment rearrangement, attenuated vessels (8 years)	Extinguished	Reduced muscle tone, psychomotor development delayed, corpus callosum hypoplasia, chronic renal failure
4–11	31/F	6 months—nystagmus, no fixation, no pacing	Light perception and projection	Hyperopic astigmatism, cataract, strong nystagmus, convergent strabismus	Thinned translucent retina, peripheral retinal pigment rearrangements, attenuated vessels, keratoconus, cataract (31 years)	Impossible to perform due to strong nystagmus	–
4–12	24/M	1 year—nystagmus, hyperopic astigmatism, strabismus	OD: 0.5/50 before, and 5/16 after corneal transplantation OS: 0.5/50	Keratoconus—now after right eye corneal transplantation (2019), keratoconus in the left eye	Drusen of the optic nerve head, retinal pigment rearrangements (14 years)	Extinguished	–
5–15	18/M	3 month—nystagmus, no fixation, no pacing	Counting fingers	High hyperopia (+ 9.0D) keratoconus, right eye corneal transplant (2016), strong nystagmus	Thinned translucent retina, peripheral retinal pigment rearrangements, attenuated vessels, loss of macular reflex (18 years)	Extinguished	–
6–18	28/F	Childhood—night blindness	Hand movements	Nystagmus (18 years), mild deterioration of color vision	Fundus: Bilateral mottled fundus, foveal atrophy with focal pigmentary changes and peripheral bone-spicule pigmentation, attenuation of the vasculature, optic nerve pallor OCT: photoreceptor loss, focal RPE hypertrophy, retinal thinning	Extinguished	–
6–19	11/M	5 month—nystagmus, childhood— night blindness	1/50 2/50	Night blindness, nystagmus	Fundus: macular atrophy, subtle pigmentary changes in the periphery OCT: photoreceptors atrophy in the macula	Extinguished	–
6–52	30/F	3 months—nystagmus, no fixation, no eye contact	Light perception and projection	Photophobia (2 years), night blindness, deterioration of color vision	Thinned translucent retina with RPE atrophy, attenuated vessels, loss of macular reflex, secondary optic nerve atrophy OCT: generalized retinal thinning, RPE hypertrophy (30 years)	Extinguished	Prolonged neonatal jaundice
7–23	12/F	1 month—nystagmus, the oculo-digital sign, absent pupillary responses	Light perception	Deep-set eyeballs	Thinned retina with salt and pepper fundus, optic disc pallor (12 years)	Extinguished	Increased muscle tone, psychomotor development delayed
7–24	15/M	After birth—nystagmus, the oculo-digital sign, absent pupillary responses	Hand movements	Hyperopic astigmatism	Thinned retina with salt and pepper fundus appearance, optic disc pallor (15 years)	Extinguished	–
8–25	6/F	2 month—nystagmus, sluggish, then absent pupillary responses, no fixation	Light perception	Hyperopia with astigmatism	Retinal pigment deposits, attenuated vessels, loss of macular reflex (2 years)	Extinguished	–
9–29	10/F	2 month—nystagmus, oculo-digital sign, photophobia, no fixation, no pacing, no eye contact	Light perception	Absent pupillary responses, nanophthalmos, hyperopia with astigmatism	Salt and pepper fundus appearance, hypoplastic optic nerve heads, attenuated vessels (6 years)	Extinguished	–
10–33	6/M	1 year—convergent strabismus	Counting fingers	Nystagmus (at 2 years), night blindness, visual field defects, hyperopic astigmatism, later myopia	Fundus, OCT, FA: thinned translucent retina with RPE atrophy, attenuated vessels, pigment deposits next to optic nerve head, diminished macular reflex (2 years)	Extinguished	–
11–36	8/M	1.5 months—nystagmus, absent pupillary responses, no fixation, no pacing	Light perception (intense light only)	–	Grey optic disc, attenuated vessels, Thinned translucent retina (3 months)	Failed to perform	–
12–39	7/M	2 months—nystagmus, profound oculo-digital sign, no fixation, no pacing, no eye contact	Light perception (intense light only)	Sluggish pupillary responses, hyperopic astigmatism (+ 6.0 D), cataract of one eye, deep-set eyeballs, still profound oculo-digital sign	OD: dense cataract (no insight to eye fundus) OS: Pale optic nerve head, loss of macular reflex, Thinned translucent retina with salt and pepper fundus appearance, attenuated vessels (6 years)	Extinguished	Reduced muscle tone, autism
13–53	20/M	2 year—nystagmus	Hand movements	Myopia and retinal degeneration at 2-year-old, sluggish pupillary responses	Funduscopy and FA: extensive atrophic retinal changes, salt and pepper fundus appearance, optic disc pallor, OCT: thinning of the retina (19 years)	Extinguished	Psychomotor development was slightly delayed,
13–54	17/M	2 months—nystagmus	Hand movements	Myopic astigmatism, sluggish pupillary responses	OCT: thinning of the retina, photoreceptors and RPE atrophy, visual field: residual (17 years)	Extinguished	Mild mental retardation
14–61	19/F	2 months—nystagmus,	1/50	Hyperopia (+ 6.0 D) strabismus	Pale optic nerve head, loss of macular reflex, retinal pigment deposits, attenuated vessels (7 years)	Extinguished	–
15–44	8/M	3 months—no fixation, no pacing, no eye contact	Hand movements	Absent pupillary responses, high hyperopia (+ 8.0 D)	Retinal pigment deposits, pale optic nerve head, loss of macular reflex (7 years)	Not performed	–
15–55	6/F	2 months—nystagmus, oculo-digital sign, no fixation, no pacing, no eye contact	Hand movements	Sluggish pupillary responses, high hyperopia	Peripheral retinal pigment deposits, pale optic nerve head, attenuated vessels, loss of macular reflex (6 years)	Extinguished	–
16–56	15/F	1 month—nystagmus, sluggish pupillary responses,	4/50 4/50	Hyperopic astigmatism, convergent strabismus, impaired night vision	Loss of macular reflex, attenuated vessels (6 years)	Extinguished	–
17–57	32/F	3 years	Light perception (intense light only)	Low-set eyeballs, visual field deficit	FA: extensive dystrophic changes; OCT: photoreceptors atrophy (9 years)	Not performed	–
18–58	42/F	3 months—nystagmus, no light perception	Light perception	Photophobia, absent pupillary responses	pigmentary changes, optic nerves atrophy (33 years)	Extinguished	–
19–47	15/M	3 months—nystagmus, strabismus	Hand movements	Hyperopic astigmatism, mild nystagmus	Macular degeneration and atrophy, optic nerves atrophy, optic disc pallor (6 years)	Extinguished	–
20–59	9/M	3 months—no fixation, no pacing, no eye contact, 4 months—nystagmus, 5 months—oculo-digital sign	No light perception	Mild nystagmus	Fundus and OCT: macular atrophy, thinned translucent and thin retina (3 years)	Extinguished	–
21–60	1.5/F	1 month—no fixation, no pacing, no eye contact, 2 months—nystagmus, 5 months—oculo-digital sign, sluggish pupillary responses	Light perception	Hyperopic astigmatism, nystagmus, oculo-digital sign	Hypoplastic, pale optic nerve head, loss of macular reflex (6 months)	scotopic response extinguished photopic—residual (7 months)	–
22–50	40/F	1 months—nystagmus and photophobia, sluggish pupillary responses	Counting fingers	Hyperopic astigmatism, photophobia, visual field constricted to 5° (38 years)	Retinal pigment deposits, esp. in the periphery (3 years)	Scotopic—diminished, photopic—extinguished (16 years)	–

*OCT* Optical Coherence Tomography, *FA* fluorescein angiography; ‘+’: present; ‘–’ not present, *BCVA* best corrected visual acuity, *OD* right eye, *OS* left eye, *M* male, *F* female

### Molecular genetic analysis

Blood samples from the affected individuals, their healthy parents, and their unaffected siblings (in families: 3, 7, 8, 12, and 19) were obtained for genetic examination (the total number of samples analyzed was 60). Genomic DNA was extracted from peripheral blood leukocytes using standard protocols. DNA samples of one proband from each family with LCA were subjected to either an LCA mutation chip based on the APEX approach or targeted NGS diagnostic panel for LCA genes (Asper Biogene, Asper Biotech Ltd., Tartu, Estonia) or both of these tests. Searching for the molecular background of LCA in patients referred to the genetic clinic until 2016 (13 families; see the last column in Table 2) was performed based on SNP microarray test, while in those who came to the clinic in 2017–2019—the NGS-LCA panel was carried out (9 families; Table 2). NGS on the diagnostic panel for LCA genes was also performed in 4 families who did not show any known variants detected by the SNP microarray. The SNP-chip for LCA was used to screen the total of 780 mutations in the following 15 genes: *AIPL1, CEP290, CRB1, CRX, GUCY2D, IQCB1, LCA5, LRAT, MERTK, RD3, RDH12, RPE65, RPGRIP1, SPATA7, TULP1.* The NGS diagnostic panel captured the following 20 LCA genes: *AIPL1, CABP4, CEP290, CRB1, CRX, GDF6, GUCY2D, IMPDH1, IQCB1, KCNJ13, LCA5, LRAT, NMNAT1, OTX2, RD3, RDH12, RPE65, RPGRIP1, SPATA7,* and *TULP1.* The panel analysis also included a position c.2991+1655A>G in the intron 26 of the *CEP290* gene. The identified sequence variants were then cross-checked to the Leiden Open Variation Database (LOVD) [4], Human Gene Mutation Database (HGMD) [5], ClinVar [6] and GnomAD browser (Genome Aggregation Database) [7]. We annotated the novel variants against the appropriate genes' reference sequences, following the Human Genome Variation Society (HGVS) nomenclature guidelines [8]. In silico analyses using SIFT (Sorting Intolerant from Tolerant) [9], PROVEAN (Protein Variation Effect Analyzer) [10] and PolyPhen-2 (Polymorphism Phenotyping v.2) [11], software were carried out to predict the possible effect of all the identified missense variants (both novel and recurrent). CADD (Combined Annotation Dependent Depletion) [12] and Fathmm (Functional Analysis Through Hidden Markov Models) [13] were additionally used to predict the possible effect of two novel splicing variants.

**Table 2:** LCA genes variants identified in Polish Patients

Family no	Mode of inheritance	Gene	Causative variations and coexisting variations				Pathogenicity prediction in protein level			Allele frequency (gnomAD browser)	Reported in literature/ variants databases: LOVD, HGMD, ClinVar	Molecular method of searching the variants
			Nucleotide	Exon/intron no	Protein	Status	SIFT	PROVEAN	PolyPhen-2			
1, 11 and 16	AR	*CEP290*	c.2991+1655A>G	i.26	p.Cys998*	Het	-	-	-	None	Yes/yes	SNP-array^1^
			c.4882C>T	e.37	p.Gln1628*	Het	-	-	-	12/172,352	Yes/yes	
2	AR	*CEP290*	c.2991+1655A>G	i.26	p.Cys998*	Het	-	-	-	None	Yes/yes	NGS
			c.4962_4963del	e.37	p.(Glu1656Asnfs*3)	Het	-	-	-	7/172,100	Yes/yes	
		*LRAT*	*c.236T<G*	e.2	*p.(Leu79Trp)*	Het	Tolerated (0.09)	Neutral (− 1.249)	Probably damaging (0.998)	1/251,328	No/no	
3	AR	*CEP290*	c.3811C>T	e.31	p.Arg1271*	Het	-	-	-	2/249,060	Yes/LOVD, HGMD	SNP-array^1^
			c.4723A>T	e.36	p.Lys1575*	Het	-	-	-	15/247,902	Yes/yes	
4	AR	*CEP290*	c.2991+1655A>G	i.26	p.Cys998*	Het	-	-	-	None	Yes/yes	NGS
			*c.6606_6618del*	e.48	p.(Ile2202Metfs*20)	Het	-	-	-	None	No/no	
5	AR	*CEP290*	c.2991+1655A>G	i.26	p.Cys998*	Het	-	-	-	None	Yes/yes	SNP-array^1^
			c.4723A>T	e.36	p.Lys1575*	Het	-	-	-	15/247,902	Yes/yes	
21	AR	*CEP290*	c.2991+1655A>G	i.26	p.Cys998*	Het	-	-	-	None	Yes/yes	NGS
			c.5515_5518del	e.40	p.(Glu1839Lysfs*11)	Het	-	-	-	3/258,898	No/LOVD, ClinVar	
8	AR	*GUCY2D*	*c.1318_1319del*	e.4	p.(Gly440Ilefs*6)	Het	-	-	-	None	No/no	NGS
			c.2302C>T	e.12	p.(Arg768Trp)	Het	Damaging (0.00)	Deleterious (− 7.478)	Probably damaging (1.0)	40/282,714	Yes/yes	
		*RPGRIP1*	*c.1414C>T*		p.(Gln472*)	Het	-	-	-	None	No/no	
12	AR	*GUCY2D*	c.2943del	e.15	p.Gly982Valfs	Het	-	-	-	None	No/ ClinVar	SNP-array^1^
			c.3118C>T	e.17	p.Arg1040Gly	Het	Damaging (0.00)	Deleterious (− 6,486)	Probably damaging (1.0)	2/237,808	Yes/HGMDClinVar	
15	AR	*GUCY2D*	c.2302C>T	e.12	p.(Arg768Trp)	Het	Damaging (0.00)	Deleterious (− 7.478)	Probably damaging (1.0)	40/282,714	Yes/yes	SNP-array^1^, NGS
			*c.721+2T>C*^a^	i.2	p.?	Het	-	-	-	None	No/no	
18	AR	*GUCY2D*	c.2943del	e.15	p.Gly982Valfs	Hom	Damaging (0.00)	Deleterious (− 7.665)	Probably damaging (1.0)	None	No/ClinVar	SNP-array^1^
6	AR	*RPE65*	*c.1451G>T*	e.14	*p.(Gly484Val)*	Hom	Damaging (0.00)	Deleterious (− 8.348)	Probably damaging (1.0)	3/248,526	No/no	NGS
10	AR	*RPE65*	c.304G>T	e.4	p.Glu102*	Hom	-	-	-	9/251,366	Yes/yes	NGS
13	AR	*RPE65*	*c.106del*	e.3	p.(Leu36Serfs*58)	Het	-	-	-	1/251,466	No/no	NGS
			*c.726-2A>T*^b^	i.7	*p.?*	Het	-	-	-	None	No/no	
9	AR	*NMNAT1*	c.59T>A	e.2	p.Ile20Asn	Het	Damaging (0.00)	Deleterious (− 5.269)	Probably damaging (0.999)	1/251,144	Yes/yes	SNP-array^1^, NGS
			c.769G>A	e.5	p.Glu257Lys	Het	Tolerated (0.72)	Neutral (− 2,313)	Benign (0.089)	196/282,064	Yes/yes	
19	AR	*NMNAT1*	*c.65A>G*	e.2	*p.(Asn22Ser)*	Het	Tolerated (0.45)	Deleterious (− 4.003)	Probably damaging (0.989)	None	No/no	NGS
			c.769G>A	e.5	p.Glu257Lys	Het	Tolerated (0.72)	Neutral (− 2.313)	Benign (0.089)	196/282,064	Yes/yes	
		*CEP290*	*c.226G<A*	e.4	*p.(Ala76Thr)*	Het	Damaging (0.00)	Neutral (− 0.560)	Probably damaging (1.0)	180/269,442	No/LOVD	
14	AR	*CRB1*	c.2843G>A	e.9	p.Cys948Tyr	Hom	Damaging (0.00)	Deleterious (− 9.655)	Probably damaging (0.998)	57/281,210	Yes/yes	NGS
17	AR	*CRB1*	c.2843G>A	e.9	p.Cys948Tyr	Hom	Damaging (0.00)	Deleterious (− 9.655)	Probably damaging (0.998)	57/281,210	Yes/yes	SNP-array^1^
7	AR	*RPGRIP1*	*c.1216del*	e.10	p.(Leu406Tyrfs*36)	Hom	-	-	-	None	No/no	SNP-array^1^, NGS
22	AR	*RPGRIP1*	*c.1148_1151del*	e.9	p.(Glu383Alafs*19)	Het	-	-	-	None	No/no	SNP-array^1^, NGS^2^
			*c.2465_2468dup*	e.16	p.(Ala824Ilefs*11)	Het	-	-	-	1/249,180	No/no	
20	AD	*CRX*	c.571delT	e.4	p.Tyr191fs*2	Het	-	-	-	None	Yes/ClinVar	SNP-array^1^

Novel variants are marked in italic

Hyphen “-”means that prediction in protein level was not performed for the variants (not necessary or improper for these variants). Allele frequency is listed according to gnomeAD Browser (Genome Aggregation Database)

^1^LCA mutation chip (SNP-microarray) based on the APEX approach (Asper Ophthalmics, Asper Biotech Ltd., Tartu, Estonia)

^2^ “Inherited Retinal Disorders NextGen Sequencing Panel” (31 genes) performed at the University of Pennsylvania at 2017

^a,b^Splicing variants submitted to additional potential pathogenicity prediction in protein level analyses with the use of CADD and Fathmm software. The results of the analyses revealed that both variants are deleterious. For the variant c.721+2T>C in the *GUCY2D* gene the CADD score is 33, and the Fathmm score is 0.97. For the variant c.726-2A>T in the *RPE65* gene the CADD score is 34 and the Fathmm score is 0.99

Segregation analysis for the presence and independent inheritance of altered alleles with Sanger sequencing of the appropriate genes fragments was performed in 17 out of 22 families, including all the families carrying the novel variants (8 families) and 9 of them with previously reported variants. The primers used for amplification and sequencing as well as the Polymerase Chain Reaction (PCR) conditions are shown in Additional file 1: Material 1. We purified the PCR products with the ExoSAP-IT kit (Exonuclease I and Shrimp Alkaline Phosphatase Cleanup for PCR products, Affymetrix) and bidirectly sequenced using dye-terminator chemistry (v3.1BigDye® Terminator, Life Technologies). The sequencing products were separated on an ABI 3130xl capillary sequencer (Applied Biosystems).

## Results

**Fig. 1:**
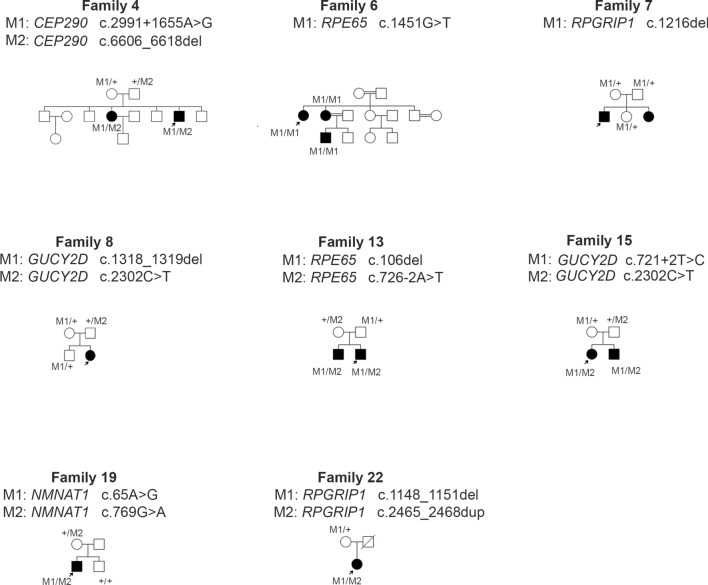
Pedigrees of the families with novel variants in LCA genes. Filled symbols indicate individuals affected with LCA and unfilled symbols indicate unaffected individuals. A slash indicates a deceased person. Arrows indicate probands

**Fig. 2:**
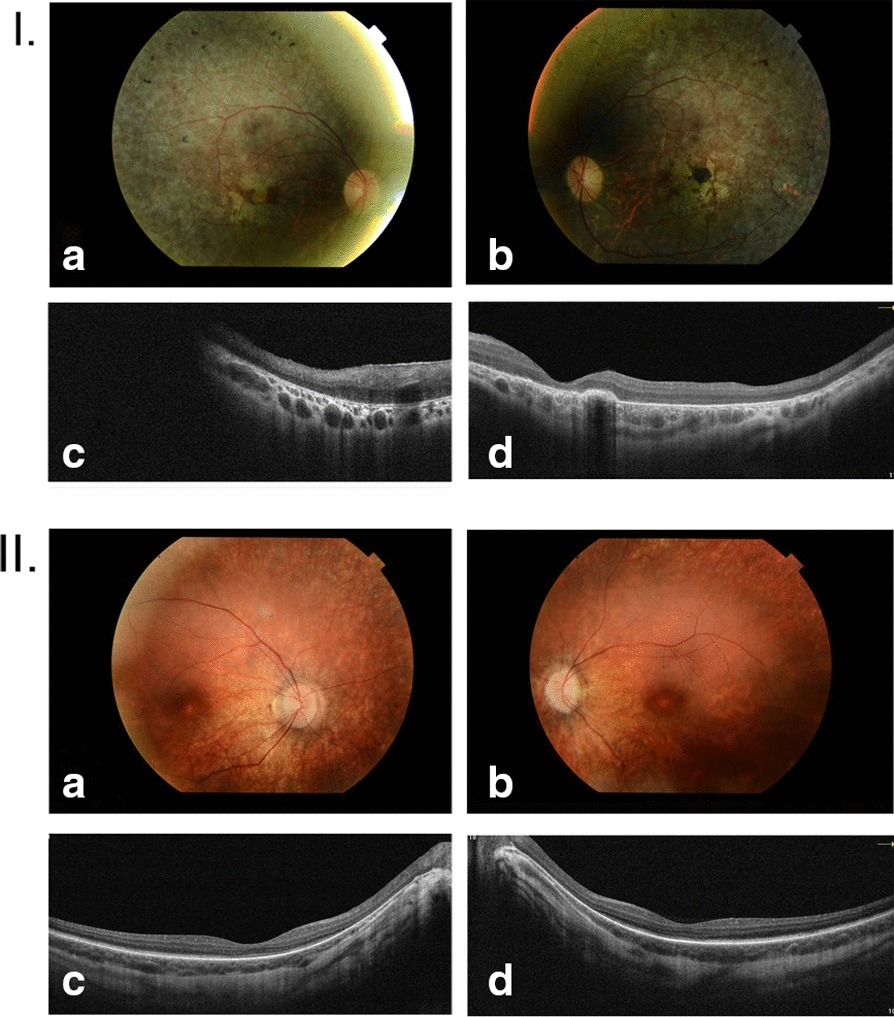
Photographs I.—Retinal features of Patient 6–18. **a**, **b** Color fundus photographs show bilateral mottled fundus appearance, foveal atrophy with focal pigmentary changes in the macula and peripheral regions (bone-spicule pigmentation), and attenuation of the vasculature and optic nerve pallor. **c** a 6-mm horizontal SD-OCT image of the right eye showing substantial photoreceptor loss, retinal architecture disorganization with thinning of outer layers, and enhanced choroidal signal penetration (the scan acquired above the fovea due to poor fixation). **d** 10 × 3.5 mm horizontal SD-OCT macular scan of the left eye demonstrating severe photoreceptor loss, focal RPE hypertrophy, and generalized retinal thinning. Photographs II.—Retinal features of Patient 13–54. **a**, **b** Color fundus images showing bilateral fine chorioretinal atrophy around the pale optic nerve with moderate vascular attenuation as well as fine peripheral pigmentary changes. **c**, **d** 10 × 3.5 mm horizontal SD-OCT scans showing intact foveal contour and symmetrical moderate thinning of outer retinal layers with enhanced choroidal signal penetration

We evaluated 28 patients from 22 unrelated families aged 1.5–42 years, exhibiting typical signs of LCA. Pedigrees of these families are shown in Fig. 1 and Additional file 2: Figure S1. Clinical symptoms and the results of the ophthalmologic examination are listed in Table 1, additionally Fig. 2 shows the retinal features of two patients.  Most of the patients presented typical symptoms for LCA: nystagmus, oculo-digital sign, no fixation, and no pacing during infancy. In some of the patients, we observed night blindness, photophobia, hyperopia, strabismus, and keratoconus. In one patient (3–7), a clinical examination performed a few years after establishing the LCA molecular diagnosis revealed some features characteristic of Joubert syndrome: chronic renal failure and psychomotor development delayed.

**Fig. 3:**
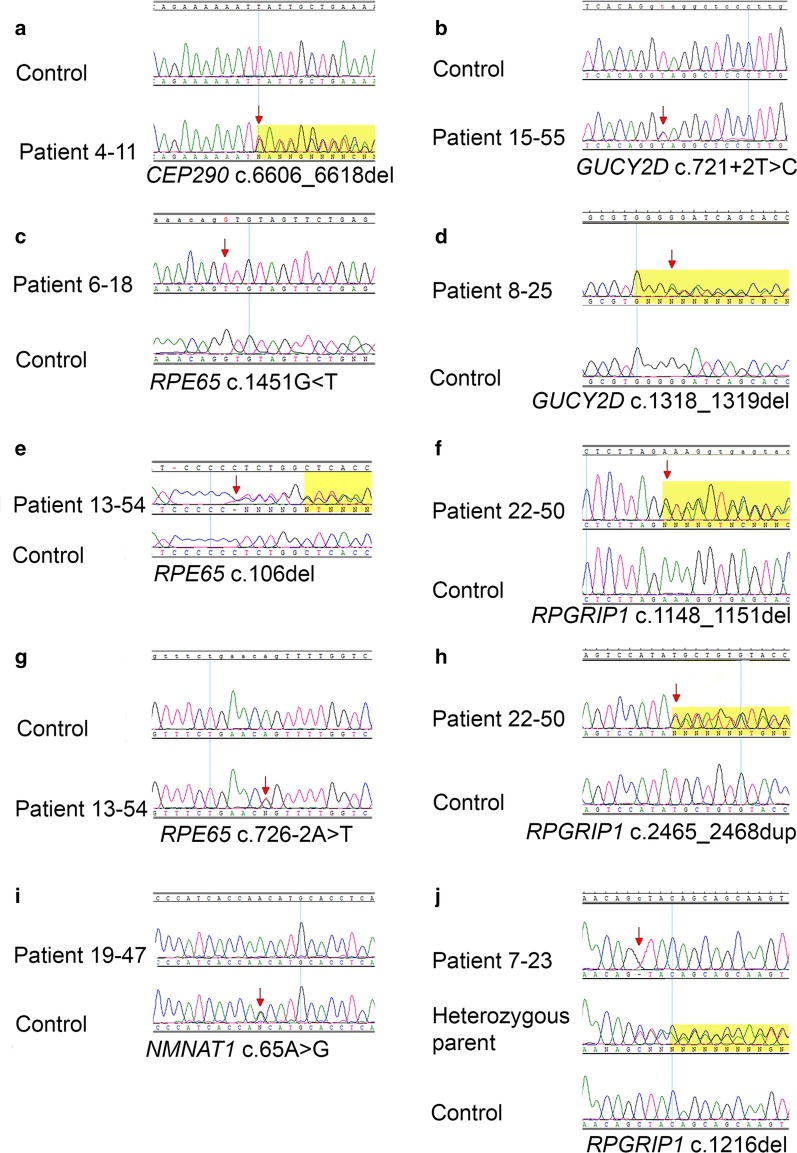
Chromatograms showing novel variants identified in LCA genes. Arrows indicate nucleotides that have been changed or the first nucleotides involved in the variation. The yellow background appears in chromatograms with frameshift variants, and it usually begins from the first nucleotide involved in the variation (excluding **d**, **e**, and **j**)

Among all the 22 examined families, 21 presented autosomal recessive inheritance patterns, while in one family, the disease appeared to have a dominant mode of inheritance. The molecular background was established in all the tested families. From a total of 24 identified variants, 23 were predicted to affect protein-coding or splicing, including 10 novel variants (see the chromatograms in Fig. 3). The variants were identified in 7 genes: *CEP290* (7 variants)*, GUCY2D* (5)*, RPE65* (4)*, NMNAT1* (3), *RPGRIP1* (3), *CRB1* (1), and *CRX* (1) (Table 2). In 8 out of 22 families, variants in the *CEP290* gene were identified. Seven families carried the substitution in the intron 26: c.2991+1655G>A (p.Cys998*). In all these individuals, the p.Cys998* variant was detected in the form of a compound heterozygote with other nonsense variants on the second allele. NGS panel for LCA genes performed in the 31-year-old female: patient 4–11 (Family 4) revealed two variants in the *CEP290* gene in the form of a compound heterozygote: a novel deletion: c.6606_6618del p.(Ile2202Metfs*20) in exon 48 and the substitution c.2991+1655G>A in the intron 26. Sanger sequencing revealed both variants' presence in the proband’s 24-year-old affected brother: patient 4–12. The deletion c.6606_6618del was not reported in the literature nor the GnomAD browser, LOVD, HGMD, and ClinVar variant databases. The phenotype of the female patient no. 4–11 was more severe than of her affected brother. Patient 4–11 had a worse vision (light perception with projection), cataract, and strong nystagmus, making it impossible to perform ERG. Patient 4–12 had a later onset of the disease with nystagmus at the age of 12 months as the first symptom. The overall course of his disease was relatively milder. He had keratoconus in both eyes. In 2019 he underwent right eye corneal transplantation, and after the surgery, his BCVA of the right eye improved significantly. The results of the SNP microarray analysis revealed that 3 patients: 1–1, 11–36, 16–54 from 3 unrelated families carry the same two *CEP290* variants: c.2991+1655G>A in the intron 26 and c.4882C>T (p.Gln1628*) in exon 37 in a compound heterozygote form. The variant p.Gln1628* is rare, and to date, it was identified as a heterozygous variant in GnomAD Browser in 1 out of 28,426 analyzed alleles in healthy individuals. NGS analysis of LCA genes performed in patient 2–4: a 3-year-old girl with severe visual impairment including reduction of visual acuity to the level of light perception, high hyperopia, nystagmus, and strabismus revealed two variants in the *CEP290* gene in a compound heterozygous state. These included the substitution in intron 26 and a deletion: c.4962_4963del in exon 37, resulting in a frameshifting: p.(Glu1656Asnfs*3). The deletion in exon 37 is a rare variant identified in a heterozygote form in GnomAD Browser. Moreover, an ultra-rare heterozygous variant in the *LRAT* gene: c.236T<G, p.(Leu79Trp) was also detected in this patient (reported in GnomAD in 1 out of 251,328 alleles in healthy individuals). The potential pathogenicity of this novel substitution is unclear. It was confirmed by the results of the in silico analyses with one (PolyPhen-2) out of three tested prediction tools, while another two (SIFT and PROVEAN) indicated that the substitution was ‘tolerated’. In the 8-year-old boy: patient 3–7 (Family 3), the SNP microarray allowed to identify two previously reported substitutions: c.3811C>T in exon 31 and c.4723A>T in exon 36, both resulted in null mutations (p.Arg1271* and p.Lys1575*, respectively). The boy presented severe visual impairment (light perception), deep-set eyeballs, and delayed psychomotor development. MRI of the head revealed corpus callosum hypoplasia. Few years after establishing the molecular diagnosis, the patient developed a chronic renal failure with cysts in renal parenchyma. The substitution c.4723A>T in exon 36 was also identified in the patient 5–15: 18-years-old man (Family 5). The results of the SNP-microarray analysis performed in this young man revealed the presence of this variant and the intronic substitution: c.2991+1655G>A. The patient had reduced visual acuity, high hyperopia, and strong nystagmus. Two *CEP290* gene variants were also identified in the patient 21–58: a 1.5-year-old girl (Family 21), with a reduction of visual acuity to the level of light perception, nystagmus, and oculo-digital sign. Panel-based NGS of LCA genes revealed a compound heterozygote of two variants: the intronic substitution: c.2991+1655G>A and 4-bp deletion c.5515_5518del in exon 40 p.(Glu1839Lysfs*11). The proband’s uncle (the brother of her mother) has also severely restricted BCVA, but the diagnosis was not confirmed (Additional file 2: Figure S1). The deletion is a rare variant described in LOVD and ClinVar databases and identified in a heterozygous state in the GnomAD browser.

In four families (8, 12, 15, and 18), the molecular analysis results revealed variants in the *GUCY2D* gene, which allowed to make a diagnosis of type 1 Leber Congenital Amaurosis (LCA1). A novel variant: 2-bp deletion c.1318_1319del in exon 4, p.(Gly440Ilefs*6) and the substitution c.2302C>T, p. (Arg768Trp) in exon 12 in the form of compound heterozygote were revealed by panel-based NGS of LCA genes in patient 8–25 (Family 8). This 6-year-old girl presented a further reduction of poor visual acuity to the level of light perception accompanied by hyperopia with astigmatism. The deletion c.1318_1319del identified in this girl was not reported in LOVD, HGMD, and ClinVar nor in the GnomAD browser, while the substitution c.2302C>T is a rare variant listed in the GnomAD browser and predicted to be probably damaging (Table 2). Moreover, in this female patient, the novel substitution: c.1414C>T (p.Gln472*) in exon 11 of *RPGRIP1* in a heterozygous state was also identified. The c.2302C>T substitution in the *GUCY2D* gene was also identified in Family 15, in the siblings: 8-year-old boy and a 6-year-old girl (15–44 and 15–53) in the form of compound heterozygote together with a novel substitution in the intron 2: c.721+2T>C. The c.2302C>T substitution was identified in the affected boy in the heterozygous state based on SNP microarray for LCA (in 2012), which at that time did not allow to establish the full molecular diagnosis. NGS panel performed in the patient's affected sister (in 2019) revealed a novel intronic variant on the second allele. The in silico predictions of the potential pathogenicity of the substitution c.721+2T>C with the use of CADD (the score: 33) and Fathmm (the score: 0.97) revealed that the variant is deleterious. Sanger sequencing of exon 12 and the fragment of the *GUCY2D* gene encompassing intronic substitution confirmed the presence of both variants in the form of compound heterozygote in both siblings. Both siblings had similar visual acuity (hand movements) and high hyperopia. Another variant in the *GUCY2D* gene identified in two Polish families (Family 12 and Family 18) was the deletion of guanine at the last nucleotide position in exon 15: c.2943del (p.Gly982Valfs). In both families, the deletion was identified based on the SNP-chip analysis for LCA. In the 42-year-old female patient 18–56 (Family 18), who had significantly reduced visual acuity (light perception) and photophobia, the c.2943del was found in a homozygous state. In the 7-year-old boy 12–39 (Family 12) with a significant reduction of visual acuity to the level of light perception, hyperopic astigmatism, and autism, the deletion in exon 15 was identified in a compound heterozygote together with the substitution c.3118C>T (p.Arg1040Gly) in exon 17. The deletion c.2943del was not reported in LOVD and HGMD, nor a control cohort annotated in the GnomAD browser. It is a rare variant reported only once in 2012 in ClinVar database without any information about its clinical significance. The in silico predictions of the potential pathogenicity of the substitution with the use of SIFT, PROVEAN, and PolyPhen-2 indicated that the variant is probably damaging. The substitution c.3118C>T (p.Arg1040Gly) is a rare variant identified in GnomAD browser in a heterozygous form in 2 out of 237,808 analyzed alleles.

*RPE65* gene variants were identified in 3 LCA families. Altogether 4 variants, including 3 novel were found. In the consanguineous family of Romany origin (Family 6), 3 out of 5 patients affected with LCA were examined in this study (Fig. 1: Pedigrees). Two sisters: no. 6–18 and 6–52 and the son of the patient no. 6–18 were referred to a genetic clinic. Two cousins of the sisters (their grandfather’s sister’s children—not examined in this study) also presented typical LCA symptoms. The parents of all the affected individuals in this family are closely related. NGS panel for LCA genes performed in a 30-year-old affected woman (6–52) revealed the presence of a novel homozygous variant: c.1451G>T in exon 14, p.(Gly484Val). Sanger sequencing revealed the homozygous substitution in exon 14 of the *RPE65* gene in two affected close relatives of the patient: her 28-year-old sister (6–18) and the 11-year-old son of this sister (6–19). In the younger sister, the onset of LCA was later, while the course of the disease was relatively milder with better visual acuity. Furthermore, she presented nystagmus from the age of 18, whereas it was observed from the age of 3 months of age in her older sister. The in silico predictions of the potential pathogenicity of the novel substitution in the *RPE65* gene with the use of SIFT, PROVEAN, and PolyPhen-2 indicated that the substitution c.1451G>T is probably damaging. The variant was not reported in the literature, nor in LOVD, HGMD and ClinVar databases, but it was identified in heterozygous state in 3 out of 248,526 alleles in healthy individuals in GnomAD Browser. In the patient 10–33 a 6-year-old boy with a reduction of visual acuity to the level of counting fingers, night blindness, astigmatism, and myopia NGS panel for LCA genes revealed a homozygous substitution c.304G>T in exon 4 (p.Glu102*) in the *RPE65* gene. This nonsense mutation is a known variant reported in LOVD and HGMD, and GnomAD Browser. In the Family 13, two novel *RPE65* variants were identified: a deletion of cytosine in exon 3: c.106del resulting in a frameshifting p.(Leu36Serfs*58) and a substitution of adenine to thymine at the 3′ end (acceptor site) of intron 7: c.726-2A>T. The in silico predictions of the potential pathogenicity of the intronic substitution with the use of CADD (the score: 34) and Fathmm (the score: 0.99) indicated that the variant is deleterious. Two brothers affected with LCA were examined: the 20-year-old man (13–53), and the 17-year-old boy (13–54). The causative variants were identified with the use of NGS for LCA genes in the older brother, and then Sanger sequencing of the appropriate *RPE65* gene fragments in the younger brother was performed, which revealed the presence of both variants. Both brothers showed a reduction of visual acuity to the level of hand movements, but the older one had a later onset of the disease (nystagmus at the age of 2 years).

LCA-type 9, characterized by severe, rapidly progressing macular degeneration with early optic nerves atrophy, were diagnosed in two families that revealed to have potentially pathogenic variants in the *NMNAT1* gene. In the patient 9–29 (Family 9): the 10-year-old girl with reduced visual acuity, nanophthalmia, and hyperopia with astigmatism, two variants in a compound heterozygote state: c.59T>A, p.(Ile20Asn) and c.769G>A, p.Glu257Lys in the *NMNAT1* were found. The substitution c.59T>A in exon 2 has been previously reported as a novel mutation in a Polish LCA patient by Falk and coworkers [14]. Substitution p.(Ile20Asn) is a rare variant identified in GnomAD browser in the form of heterozygote in 1 out of 121,376 analyzed alleles. The in silico predictions of the potential pathogenicity of the substitution with the use of SIFT, PROVEAN, and PolyPhen-2 indicated that the variant is probably damaging (for the scores see Table 2). In the patient 47–19 (Family 19): the 15-year-old boy with a reduction of visual acuity to the level of hand movements, hyperopic astigmatism, and mild nystagmus, panel-based NGS of LCA genes revealed a compound heterozygote of two variants in the *NMNAT1*: a novel substitution c.65A>G, p.(Asn22Ser) in exon 2 and a known variant in exon 5: c.769G>A. The variant c.65A>G was not reported in the literature nor the GnomAD browser. This new substitution p.(Asn22Ser) can be considered as a pathogenic variant according to the results of two out of three in silico pathogenicity prediction tools tested in this study: PolyPhen-2 (probably damaging) and PROVEAN (deleterious). In contrast, SIFT results suggested that it is predicted not to damage the protein function (tolerated) (Table 2). The variant c.769G>A is a frequent substitution reported in GnomAD browser in the form of heterozygote in 74 out of 120,500 analyzed alleles. The results of the in silico predictions suggest that the p.Glu257Lys variant's pathogenicity is doubtful (SIFT prediction indicated that would be tolerated, PROVEAN—neutral, while PolyPhen-2—benign). The NGS analyses results also revealed a heterozygous substitution: c.226G>A p.(Ala76Thr) in the *CEP290* gene.

Based on the results of molecular analysis LCA-type 6 was diagnosed in two Polish families. Altogether three variants in *RPGRIP1* gene were identified. All of them were novel. A new homozygous variant was identified in two affected children in the Family 7: a 12-years-old girl (7–23) and a 15-years-old boy (7–24). Both children have similar symptoms and fundus appearance, but the boy has better visual acuity (hand movements) than the girl (light perception). SNP microarray performed in both children did not identify any potentially pathogenic variants in LCA genes, but NGS on LCA panel conducted in the boy a few years later revealed a homozygous, novel deletion c.1216del, p.(Leu406Tyrfs*36) in exon 10 in *RPGRIP1* gene. Sanger sequencing of the *RPGRIP1* exon 10 showed the same variant: a guanine deletion at the nucleotide position 1216 in a homozygous state in the affected sister. The variant was not reported in LOVD and HGMD, nor a control cohort annotated in the GnomAD browser. Moreover, NGS analysis identified a heterozygous variant in the *GUCY2D* gene: a substitution: c.262C>A p.(Pro88Thr) in exon 2. *RPGRIP1* variants were also identified in patient 22–50 (Family 22)—the 40-year-old woman with a reduction of visual acuity to the level of counting fingers, photophobia, and hyperopic astigmatism. SNP microarray did not identify any potentially pathogenic variants in LCA genes, but NGS on the LCA panel revealed two novel frameshift *RPGRIP1* variants: c.1148_1151del in exon 9 p.(Glu383Alafs*19) and c.2465_2468dup in exon 16 p.(Ala824Ilefs*11). The variant p.(Glu383Alafs*19) was not reported in LOVD and HGMD, nor in a control cohort annotated in the GnomAD browser, while the p.(Ala824Ilefs*11) is a rare variant identified in GnomAD browser in the form of heterozygote in 1 out of 249,180 analyzed alleles.

In two families (14 and 17), the molecular analysis results revealed the presence of a homozygous variant in the *CRB1* gene, which allowed to make a diagnosis of LCA8. In both families, a homozygous substitution c.2843G>A in exon 9 was identified (p.Cys948Tyr). In a patient no. 14–61: the 19-year-old female patient with visual acuity 1/50, hyperopia, and strabismus, this known variant was identified based on NGS analysis for LCA genes, while in the patient no. 17–57: the 32-year-old woman with poor vision (light perception) was detected using SNP-array.

In 1 out of 22 families, the disease has a dominant mode of inheritance. In the patient 20–57 (Family 20), a completely blind (no light perception) 9-year-old boy, a heterozygous deletion c.571delT (p.Tyr191fs*2) in the *CRX* gene was identified based on SNP microarray analysis for LCA genes. The deletion is de novo variant, as it was not identified in both healthy parents of the proband. The variant was not reported in LOVD and HGMD, nor a control cohort annotated in the GnomAD browser.

Segregation analysis for the presence and independent inheritance of altered alleles with Sanger sequencing of the appropriate genes fragments was performed in altogether 17 out of 22 families, including all the families carrying the novel variants (8 families) and 9 of those with previously reported variants. In most families, segregation analysis was performed in both parents of the proband/probands (excluding parents of the sisters 6–18 and 6–52; deceased father of the patient 22–50 and the father of the patient 19–47 who was impossible to involve in the study due to his serious cancer). Moreover, in some families, this analysis was also performed in healthy siblings of the probands. The segregation analysis was impossible to perform in 5 families: 14, 16, 17, 18, 21, due to parents' inaccessibility or lack of consent for testing. The results of the segregation analysis were consistent with the Mendelian inheritance (AR in most families and AD with a de novo variant in one family: Family 20).

## Discussion

In the studied group of 22 Polish families suffering from LCA, 24 variants in 7 genes, including 10 novel, were identified. LCA-10 type appeared to be the most common form of the disease, as variants in the *CEP290* gene were revealed in 8 out of 22 families, representing 36%. The most commonly detected variant was the substitution in the intron 26: c.2991+1655G>A (p.Cys998*) identified in 7 families, which accounts for one-third of all examined families. It is consistent with the determination of this variant as the most common pathogenic mutation in the *CEP290* gene, especially in Europe and the United States [15]. In all the affected individuals with the p.Cys998* variant, this mutation was detected in the form of a compound heterozygote with other nonsense variants on the second allele. Therefore, all the *CEP290* variants identified in this study encode premature stop codons (Table 2), which would lead to protein truncation and the loss of critical functional domains. Missense variants of the *CEP290* are rare (LOVD, ClinVar), and probably they are well-tolerated and do not sufficiently abrogate CEP290 protein and function. Patients with two nonsense variants appear to have worse visual acuity than patients with one nonsense and one missense *CEP290* variant [15, 16]. In a cohort of German patients with *CEP290* variants, it has been observed that homozygous patients for the c.2991+1655G>A variant presented a more severe phenotype than compound heterozygotes with this intronic variant [17]. In our study group, it was difficult to observe any genotype–phenotype correlations similar to those presented in a German patient group, as there were no homozygotes for the variant p.Cys998* in our group of patients. Moreover, even in the group of patients with the same two *CEP290* variants, for example, three families (no. 1, 11, 16) carrying c.2991+1655G>A and c.4882C>T (p.Gln1628*) in exon 37 it was impossible to compare the phenotypes of the individuals as they are of different ages and we did not have retrospective data. The finding that the variant c.4882C>T (p.Gln1628*), which is described as rare in GnomAD browser was identified in 3 out of 8 Polish families with *CEP290* gene affected is intriguing and suggests that is a common variant in Polish LCA patients.

Apart from isolated blindness, mutations in the *CEP290* gene can cause various syndromes like Bardett-Biedl syndrome [18], Meckel-Gruber syndrome [19], Senior-Loken syndrome, and Joubert syndrome [20]. In our group of patients with *CEP290* variants, non-ocular symptoms were observed only in two patients. Patient 2–4 carrying a deletion in exon 37: c.4962_4963del and a common substitution in the intron 26 had muscular hypotonia after birth. Patient 3–7, a compound heterozygote of two substitutions: c.3811C>T and c.4723A>T, had reduced muscle tone, delayed psychomotor development, and developed a chronic renal failure with cysts in renal parenchyma about 6 years after establishing the diagnosis of LCA. The boy did not present any face dysmorphic features. MRI revealed corpus callosum hypoplasia, but it did not show characteristics for Joubert syndrome-related disorders (JSRDs) brain abnormality: the so-called ‘molar tooth sign’ (MTS). MTS is an abnormal development of the cerebellar vermis and the brainstem resembling the cross-section of a molar tooth in brain imaging. As this brain disorder considered a hallmark for JSRDs diagnosis is not present in the patient 3–7, Joubert syndrome's oculo-renal form was not recognized. Both substitutions: the c.3811C>T and the frequent: c.4723A>T identified in our patient have been reported in patients with isolated LCA and other retinal dystrophies but also in patients showing retinal disorders with MTS [15, 17, 20, 21]. The substitution c.4723A>T (p.Lys1575*) identified in the patient no. 3–7 was also found in the patient no. 5–15. Both male patients from these two families have similar visual acuity (light perception) and shared common LCA symptoms like nystagmus, oculo-digital sign, and hyperopia. However, the results of the patient’s 5–15 head MRI examination and psychomotor development were normal, and he did not present any renal failures like patient 3–7. The phenotype of the 31-year-old female patient no. 4–11 with a novel variant c.6606_6618del, p.(Ile2202Metfs*20) of *CEP290* was more severe than her 24-year-old brother. The deletion c.6606_6618del causes a frameshift and an introduction of the premature stop codon in the last coiled-coil domain of the CEP290 protein. This domain is also a part of the myosin-tail (MYO-tail) homology domain [22]. The MYO-tail homology domain's presence may provide a structural backbone to the myosin motor and could facilitate the microtubule-associated transport of CEP290 to centrosomes [23]. This C-terminal domain is essential for protein confinement between the inner and outer segments in photoreceptors [24].

LCA1 was diagnosed in four families. Altogether five variants (including two novel ones) in *GUCY2D* gene were found. The novel variant c.1318_1319del p.(Gly440Ilefs*6) identified in the patient 8–25 produced premature stop at the 446 codon, which plausibly caused the production of the truncated protein lacking the entire intracellular domain, including the protein homology region and C-terminal catalytic domain [25]. Three children (from families 8 and 15) with the substitution c.2302C>T have similar clinical symptoms, but two siblings 15–44 and 15–53, who also carried a novel intronic variant (c.721+2T>C) appear to have better visual acuity (hand movements) than the girl with a novel nonsense mutation: p.(Gly440Ilefs*6) on the second allele (light perception). The novel splicing variation c.721+2T>C was predicted to be deleterious. The slight differences in children's phenotypes from these two families might be explained by the fact that splicing variants probably cause milder defects than nonsense variants. Moreover, the patient 8–25 also carried a novel substitution: c.1414C>T (p.Gln472*) in exon 11 of *RPGRIP1* in a heterozygous state. According to the STRING database [26] RPGRIP1 protein is not a predicted functional partner of GUCY2D, but we cannot exclude this variant as a modifying factor of the disease. The rare deletion c.2943del (p.Gly982Valfs), as far as we know reported to date only in one family, was identified in two families examined in this study: in the patient 12–39 and the patient 18–58. It is difficult to compare the phenotypes of these patients as they are of different ages (7-year-old boy and 42-year-old woman, respectively), and we did not have full data about the course of the disease in the infancy in the female patient. Studies on a group of 21 patients with molecularly confirmed LCA1 revealed the relatively preserved photoreceptor structure over a broad age range indicating a wide therapeutic window for gene therapy trials [27].

Altogether 4 *RPE65* variants, including 3 novel ones, were identified in 3 LCA2 families. Two new variants were identified in two affected brothers. The frameshift variant c.106del produced premature stop codon p.(Leu36Serfs*58) at the amino acid position 94 of the RPE65 protein. Therefore, because the wild-type protein was composed of 533 amino acids, it was likely to be a null allele. The novel splicing variation c.726-2A>T was predicted to be deleterious. The novel homozygous substitution c.1451G>T (p.Gly484Val) localized in the last exon of the *RPE65* gene was identified in the consanguineous family of Romany origin. The homozygous substitution c.1451G>A resulting in p.Gly484Asp was previously reported in two male patients with a severe form of LCA [28]. The amino acid glycine at the residue 484 is highly conserved between species [29], and this missense variant p.Gly484Val is predicted by the in silico analyses to affect protein function, although functional analyses are required to elucidate the pathogenicity of this substitution. Visual acuity varies between patients with different *RPE65* genotypes, but also between the members of the same family examined in this study (from 1/50 to light perception and projection in Family 6). It is difficult to find any genotype–phenotype correlation in such a small patient group, as visual acuity also correlates with the patient’s age.

LCA 9 was identified in 2 out of 22 families. Among three variants identified in the *NMNAT1* gene one was novel. The potential pathogenicity of the novel substitution c.65A>G, p.(Asn22Ser) was suggested by the results of the in silico analyses with the use of two prediction tolls: PROVEAN and PolyPhen, but as SIFT results indicated the substitution is ‘tolerated’, it remains unclear and requires to be elucidated by the functional analyses. The substitution c.59T>A identified in patient 9–29 is an ultra-rare variant that has been previously reported as a novel mutation in one Polish patient with LCA by Falk and coworkers. The female patient described by Falk and coworkers [14] has the same genotype as our patient: she is a compound heterozygote of two substitutions: c.59T>A and a c.769G>A. The phenotype of the patient, reported by Falk, was also similar to that presented by our patient 9–29. Both girls presented a reduction of visual acuity to the level of light perception, nystagmus, oculo-digital sign, and hyperopia. This ultra-rare variant may be present only in Polish patients. The substitution c.769G>A (p.Glu257Lys) identified in two Polish families is the most frequently observed *NMNAT1* variant, accounting for more than 70% of LCA9 cases based on previously published reports [14, 30–34]. Considering the high population frequency of the variant p.Glu257Lys, the results of the in silico pathogenicity predictions suggesting that it may be non-pathogenic, and the fact that the homozygous substitution was also identified in individuals with no ocular phenotypes [34, 35], the variant's pathogenicity has been questioned. Therefore, recently the analyses on the knock-in mouse model were performed. The homozygous mice Nmnat1^E257K/ E257K^ did not exhibit any retinal phenotype. The compound heterozygous mice Nmnat1^E257K/−^ generated by crossing mice with a heterozygous deletion of exon 2 Nmnat1^−/+^ with mice Nmnat1^E257K/E257K^ appeared to be normal without any retinal phenotype but had thinned photoreceptor layer at 5 months age. The detection of the activated ER stress markers expression in the retina after intense light exposure suggested that the Nmnat1^E257K/−^ mice are more susceptible to ER stress, which likely contributes to photoreceptor degeneration and death [34]. The functional analyses revealed that the p.Glu257Lys variant results in reduced enzymatic activity and altered the protein's structural stability under stress condition [31, 36].

Three novel *RPGRIP1* variants were identified: a homozygous deletion c.1216del p.(Leu406Tyrfs*36) in the affected siblings from Family 7, and two frameshift variants: c.1148_1151del p.(Glu383Alafs*19) and c.2465_2468dup p.(Ala824Ilefs*11) in a female patient from Family 22. All these frameshift variants produce premature stop codons, which most likely result in a truncated protein. The variants p.(Glu383Alafs*19) and p.(Leu406Tyrfs*36) are localized in the N-terminal part of the protein, while p.(Ala824Ilefs*11) is localized in a central part of the RPGRIP1 protein—in a conserved region 2 (C2) domain. C2 domains are implicated in Ca2-dependent membrane docking of proteins and in mediating protein–protein interactions [37]. The symptoms in early childhood were very similar in all these three patients with *RPGRIP1* variants, but the visual acuity is better in the oldest patient: 22–50.

In two genes: *CRB1* and *CRX,* only known variants were identified in the studied group. A homozygous substitution c.2843G>A (p.Cys948Tyr) in the *CRB1* gene was identified in two women from two nonconsanguineous families: 19-year-old and 32-years-old, with worse vision in the older female patient. In 1 out of 22 families, the disease has a dominant mode of inheritance. The heterozygous deletion c.571delT (p.Tyr191fs*2) in the *CRX* gene identified in a totally blind boy is de novo variant, as it was not identified in both healthy parents of the proband. The variant was predicted to encode the mutant form of CRX with altered carboxy termini. To the best of our knowledge, the deletion was previously described in one patient with LCA [38].

All the examined patients with LCA underwent funduscopy, some of them also had SD-OCT or SD-OCT and AF performed. There was no correlation between the fundus appearance and the gene in which potentially pathogenic variants were identified. The identification of causative variants in 7 different genes in a relatively small study group of 22 LCA families together with the fact that patients were at a different age, and sometimes they lack the results of ophthalmologic examination from early childhood, make difficult to follow any phenotype-genotype correlations.

The distribution of pathogenic genes varies considerably among different populations of patients with LCA [33, 39, 40]. In the studied group of Polish patients, mutations in three genes (*CEP290*, *GUCY2D,* and *RPE65*) that are currently the targets for gene therapy appeared to be the most frequent cause of the disease. This observation strongly emphasizes the importance and the need for molecular background analyses in LCA in Polish patients, the results of which can directly contribute to enabling treatment with gene therapy. Genetic analysis should be performed at the early stages of the disease as some gene therapies may need to be given in infancy to achieve the best visual outcome [15]. Moreover, it is crucial to conduct a gene-editing approach before photoreceptors have totally degenerated. The problem is that the accurate clinical diagnosis of the child affected with retinal dystrophy is sometimes challenging to establish at the first visit, which often hampers the choice of the appropriate targeted genetic analysis.

In this study, we report LCA families referred to the genetic clinic in 2010–2019. Until 2016 SNP microarray for LCA genes was considered as the best available method. Then, in 2017 NGS panel for LCA genes replaced this method, and from that time, NGS panel has been offered to all patients with suspected LCA referred to our genetic clinic. Nowadays, NGS panel is still a more commonly performed method to search for the molecular background of LCA, but considering the decreasing cost of WES (Whole Exome Sequencing), the WES approach is performed as a method of choice in more and more cases, especially in those with an unclear diagnosis. Awareness of the genetic analysis importance and cooperation between ophthalmologists and geneticists cannot be overestimated in making an accurate clinical diagnosis and planning treatment. Leber congenital amaurosis type 2 was the first human monogenic retinal disorder tested for ocular gene therapy. Subretinal surgical delivery of live non-replication adenoviral vector carrying *RPE65* gene (Voretigene Neparvovec-rzyl—brand name: Luxturna™) provides healthy human RPE65 protein to some RPE cells, which makes possible to restore the visual cycle. Gene therapy is available for patients with biallelic *RPE65* mutations and viable retinal cells, as it was approved and registered in 2017 [41]. This therapy has an acceptable safety profile. However, the recent meta-analysis summarizing the results of six clinical trials, including 164 eyes, showed that efficacy in improving best-corrected visual acuity appears to be limited to 2 years after treatment [42]. Moreover, some tendency for thinning of the RPE layer faster in the treated eye than in the non-treated one was observed even in the first year after treatment. However, there is a hope to prolong the efficacy of the therapy. It is suggested to administer combinatorial agents supplementing the gene therapy to slow retinal degeneration in the long term [42, 43].

The possibility of LCA treatment has emerged recently. Clinical trials are underway in patients with LCA caused by a frequent intronic variant c.2991+1655A>G in *CEP290* gene. The treatment results (phase 1 and 2) based on intravitreal injections of antisense oligonucleotides (QR-110) to restore correct splicing are promising. In 10 treated patients, there were no serious adverse effects, and visual acuity improved after 3 months of treatment [44]. Moreover, a specific strategy based on the gene-editing approach has emerged recently for the c.2991+1655A>G *CEP290* intronic variant. A candidate genome-editing therapeutic: EDIT-101 uses an AAV5 vector to deliver the Staphylococcus aureus Cas9 and CEP290-specific guide RNAs (gRNAs) to photoreceptor cells by subretinal injection. The injection was well tolerated and allowed to achieve sustained and dose-dependent CEP290 editing in photoreceptor cells, in mice and non-human primates [45]. Gene replacement therapy will be tested in patients with LCA caused by biallelic mutations in *GUCY2D* gene. The subretinal injection of the adenoviral vector carrying GUCY2D gene (SAR439483) will be performed in 15 patients to evaluate this therapy's safety and tolerability [https://clinicaltrials.gov/].

A gene replacement attempts on a mouse model with retinal degeneration were performed for *NMNAT1* and *CRB1* genes, also associated with LCA in a studied group of Polish patients*.* Subretinal injection of a normal copy of human *NMNAT1* via adeno-associated virus (AAV) into mice resulted in the preservation of retinal structure and function for at least 9 months [46]. Human *CRB2* that is the *CRB1* preferable substitute, was targeted in AAV both to Müller glial and photoreceptors into the retinitis pigmentosa mouse model, which ameliorated the retinal function and structure [47]. Numerous completed and ongoing gene replacement studies on animal models give hope for clinical trials in humans in the near future and effective treatment of more LCA forms.

## Conclusions

This study provides the first molecular genetic characteristics of patients with Leber congenital amaurosis from the previously unexplored Polish population. Our study expands the mutational spectrum as we report 10 novel variants identified in LCA genes. The most frequent causes of the disease in the studied group of Polish patients are mutations in one out of three genes that are currently the targets for gene therapy (*CEP290*, *GUCY2D,* and *RPE65*), which strongly emphasizes the importance of the molecular background analyses of LCA in Polish patients.

## Supplementary Information

**Additional file 1**. **Material 1.** Primer pairs and size of PCR products used for Sanger sequencing in this study.

**Additional file 2**. **Figure S1.** Pedigrees of the families with known variants in LCA genes. Black filled symbols indicate individuals affected with LCA, unfilled symbols indicate unaffected individuals, while grey filled square indicates a patient with undefined vision disorders. A slash indicates a deceased person. Arrows indicate probands.

## Data Availability

Not applicable. Data sharing not applicable to this article as no datasets were generated or analyzed during the current study.

## Abbreviations

APEX: Arrayed Primer Extension
ARVO: Association for Research in Vision and Ophthalmology
BCVA: Best-corrected visual acuity
CADD: Combined Annotation Dependent Depletion
ERG: Electroretinography
FA: Fluorescein angiography
Fathmm: Functional Analysis Through Hidden Markov Models (v2.3)
gnomAD browser: Genome aggregation database
HGMD: Human Gene Mutation Database
HGVS: Human Genome Variation Society
IRD: Inherited retinal dystrophies
LCA: Leber congenital amaurosis
LOVD: Leiden Open Variation Database
MRI: Magnetic resonance imaging
NGS: Next Generation Sequencing
PCR: Polymerase Chain Reaction
Polyphen-2: Polymorphism Phenotyping v2
PROVEAN: Protein Variation Effect Analyzer
SD-OCT: Spectral-Domain Optical Coherent Tomography
SIFT: Sorting Intolerant from Tolerant
SNP: Single Nucleotide Polymorphism
WES: Whole Exome Sequencing

## References

[1] Koenekoop RK, Lopez I, den Hollander AI, Allikmets R, Cremers FP (2007). Genetic testing for retinal dystrophies and dysfunctions: benefits, dilemmas and solutions. Clin Exp Ophthalmol.

[2] Weleber RG, Francis PJ, Trzupek KM, Beattie C. Leber Congenital Amaurosis.2004 Jul 7 [updated 2013 May 2]. In: Adam MP, Ardinger HH, Pagon RA, Wallace SE, Bean LJH, Stephens K, Amemiya A, editors. GeneReviews® [Internet]. Seattle (WA): University of Washington, Seattle. 1993–2019. http://www.ncbi.nlm.nih.gov/books/NBK1298/PubMed.

[3] RetNet. https://sph.uth.edu/retnet/. Accessed 16 June 2020.

[4] Leiden Open Variation Database (LOVD). http://www.lovd.nl/3.0/home. Accessed 10 Apr 2020.

[5] Human Genome Mutation Database (HGMD). http://www.hgmd.cf.ac.uk/ac/index.php. Accessed 10 Apr 2020.

[6] ClinVar. https://www.ncbi.nlm.nih.gov/clinvar/. Accessed 10 Apr 2020.

[7] Genome aggregation database (gnomAD browser). https://gnomad.broadinstitute.org/. Accessed 10 Apr 2020.

[8] Human Genome Variation Society (HGVS). http://varnomen.hgvs.org/. Accessed 10 Apr 202.

[9] Sorting Intolerant From Tolerant (SIFT). https://sift.bii.a-star.edu.sg/. Accessed 10 Apr 2020.

[10] Protein Variation Effect Analyzer (PROVEAN). http://provean.jcvi.org/. Accessed 10 Apr 2020.

[11] Polymorphism Phenotyping v2 (Polyphen-2). http://genetics.bwh.harvard.edu/pph2/. Accessed 10 Apr 2020.

[12] Combined Annotation Dependent Depletion (CADD). https://cadd.gs.washington.edu/snv. Accessed 10 Apr 2020.

[13] Functional Analysis through Hidden Markov Models (v2.3) (Fathmm). http://fathmm.biocompute.org.uk/fathmm-xf/. Accessed 10 Apr 2020.

[14] Falk MJ, Zhang Q, Nakamaru-Ogiso E, Kannabiran C, Fonseca-Kelly Z, Chakarova C, Audo I, Mackay DS, Zeitz C, Borman AD, Staniszewska M, Shukla R, Palavalli L, Mohand-Said S, Waseem NH, Jalali S, Perin JC, Place E, Ostrovsky J, Xiao R, Bhattacharya SS, Consugar M, Webster AR, Sahel JA, Moore AT, Berson EL, Liu Q, Gai X, Pierce EA (2012). NMNAT1 mutations cause Leber congenital amaurosis. Nat Genet.

[15] Sheck L, Davies WIL, Moradi P, Robson AG, Kumaran N, Liasis AC, Webster AR, Moore AT, Michaelides M (2018). Leber congenital amaurosis associated with mutations in CEP290, clinical phenotype, and natural history in preparation for trials of novel therapies. Ophthalmology.

[16] Yzer S, Hollander AI, Lopez I, Pott JW, de Faber JT, Cremers FP, Koenekoop RK, van den Born LI (2012). Ocular and extra-ocular features of patients with Leber congenital amaurosis and mutations in CEP290. Mol Vis.

[17] Feldhaus B, Weisschuh N, Nasser F, den Hollander AI, Cremers FPM, Zrenner E, Kohl S, Zobor D (2020). CEP290 mutation spectrum and delineation of the associated phenotype in a large German cohort: a monocentric study. Am J Ophthalmol.

[18] Leitch CC, Zaghloul NA, Davis EE, Stoetzel C, Diaz-Font A, Rix S, Alfadhel M, Lewis RA, Eyaid W, Banin E, Dollfus H, Beales PL, Badano JL, Katsanis N. Hypomorphic mutations in syndromic encephalocele genes are associated with Bardet-Biedl syndrome. Nat Genet. 2008; 40(4):443–8. Erratum in: Nat Genet. 2008; 40(7):927. Al-Fadhel, Majid [corrected to Alfadhel, Majid]. 10.1038/ng.97 18327255

[19] Baala L, Audollent S, Martinovic J, Ozilou C, Babron MC, Sivanandamoorthy S, Saunier S, Salomon R, Gonzales M, Rattenberry E, Esculpavit C, Toutain A, Moraine C, Parent P, Marcorelles P, Dauge MC, Roume J, Le Merrer M, Meiner V, Meir K, Menez F, Beaufrère AM, Francannet C, Tantau J, Sinico M, Dumez Y, MacDonald F, Munnich A, Lyonnet S, Gubler MC, Génin E, Johnson CA, Vekemans M, Encha-Razavi F, Attié-Bitach T (2007). Pleiotropic effects of CEP290 (NPHP6) mutations extend to Meckel syndrome. Am J Hum Genet.

[20] Brancati F, Barrano G, Silhavy JL, Marsh SE, Travaglini L, Bielas SL, Amorini M, Zablocka D, Kayserili H, Al-Gazali L, Bertini E, Boltshauser E, D'Hooghe M, Fazzi E, Fenerci EY, Hennekam RC, Kiss A, Lees MM, Marco E, Phadke SR, Rigoli L, Romano S, Salpietro CD, Sherr EH, Signorini S, Stromme P, Stuart B, Sztriha L, Viskochil DH, Yuksel A, Dallapiccola B; International JSRD Study Group, Valente EM, Gleeson JG. CEP290 mutations are frequently identified in the oculo-renal form of Joubert syndrome-related disorders. Am J Hum Genet. 2007; 81(1):104–13. 10.1086/519026 PMC1950920 17564967

[21] Perrault I, Delphin N, Hanein S, Gerber S, Dufier JL, Roche O, Defoort-Dhellemmes S, Dollfus H, Fazzi E, Munnich A, Kaplan J, Rozet JM (2007). Spectrum of NPHP6/CEP290 mutations in Leber congenital amaurosis and delineation of the associated phenotype. Hum Mutat.

[22] Moradi P, Davies WL, Mackay DS, Cheetham ME, Moore AT (2011). Focus on molecules: centrosomal protein 290 (CEP290). Exp Eye Res.

[23] Chang B, Khanna H, Hawes N, Jimeno D, He S, Lillo C, Parapuram SK, Cheng H, Scott A, Hurd RE, Sayer JA, Otto EA, Attanasio M, O’Toole JF, Jin G, Shou C, Hildebrandt F, Williams DS, Heckenlively JR, Swaroop A (2006). In-frame deletion in a novel centrosomal/ciliary protein CEP290/NPHP6 perturbs its interaction with RPGR and results in early-onset retinal degeneration in the rd16 mouse. Hum Mol Genet.

[24] Datta P, Hendrickson B, Brendalen S, Ruffcorn A, Seo S (2019). The myosin-tail homology domain of centrosomal protein 290 is essential for protein confinement between the inner and outer segments in photoreceptors. J Biol Chem.

[25] Shyjan AW, de Sauvage FJ, Gillett NA, Goeddel DV, Lowe DG (1992). Molecular cloning of a retina-specific membrane guanylyl cyclase. Neuron.

[26] Functional protein association network STRING database. https://string-db.org/.

[27] Bouzia Z, Georgiou M, Hull S, Robson AG, Fujinami K, Rotsos T, Pontikos N, Arno G, Webster AR, Hardcastle AJ, Fiorentino A, Michaelides M (2020). GUCY2D-associated Leber congenital amaurosis: a retrospective natural history study in preparation for trials of novel therapies. Am J Ophthalmol.

[28] Kumaran N, Rubin GS, Kalitzeos A, Fujinami K, Bainbridge J, Weleber RG, Michaelides M (2018). A cross-sectional and longitudinal study of retinal sensitivity in RPE65-associated Leber Congenital amaurosis. Investig Ophthalmol Vis Sci.

[29] Takahashi Y, Moiseyev G, Ma JX (2014). Identification of key residues determining isomerohydrolase activity of human RPE65. J Biol Chem.

[30] Chiang PW, Wang J, Chen Y, Fu Q, Zhong J, Chen Y, Yi X, Wu R, Gan H, Shi Y, Chen Y, Barnett C, Wheaton D, Day M, Sutherland J, Heon E, Weleber RG, Gabriel LA, Cong P, Chuang K, Ye S, Sallum JM, Qi M (2012). Exome sequencing identifies NMNAT1 mutations as a cause of Leber congenital amaurosis. Nat Genet.

[31] Koenekoop RK, Wang H, Majewski J, Wang X, Lopez I, Ren H, Chen Y, Li Y, Fishman GA, Genead M, Schwartzentruber J, Solanki N, Traboulsi EI, Cheng J, Logan CV, McKibbin M, Hayward BE, Parry DA, Johnson CA, Nageeb M, Finding of Rare Disease Genes Canada C; Poulter JA, Mohamed MD, Jafri H, Rashid Y, Taylor GR, Keser V, Mardon G, Xu H, Inglehearn CF, Fu Q, Toomes C, Chen R. Mutations in NMNAT1 cause Leber congenital amaurosis and identify a new disease pathway for retinal degeneration. Nat Genet. 2012; 44:1035–9. 10.1038/ng.2356 PMC3657614 22842230

[32] Perrault I, Hanein S, Zanlonghi X, Serre V, Nicouleau M, Defoort-Delhemmes S, Delphin N, FaresTaie L, Gerber S, Xerri O, Edelson C, Goldenberg A, Duncombe A, Le Meur G, Hamel C, Silva E, Nitschke P, Calvas P, Munnich A, Roche O, Dollfus H, Kaplan J, Rozet JM (2012). Mutations in NMNAT1 cause Leber congenital amaurosis with early-onset severe macular and optic atrophy. Nat Genet.

[33] Thompson JA, De Roach JN, McLaren TL, Montgomery HE, Hoffmann LH, Campbell IR, Chen FK, Mackey DA, Lamey TM (2017). The genetic profile of Leber congenital amaurosis in an Australian cohort. Mol Genet Genomic Med.

[34] Eblimit A, Zaneveld SA, Liu W, Thomas K, Wang K, Li Y, Mardon G, Chen R (2018). NMNAT1 E257K variant, associated with Leber Congenital Amaurosis (LCA9), causes a mild retinal degeneration phenotype. Exp Eye Res.

[35] Siemiatkowska AM, Schuurs-Hoeijmakers JH, Bosch DG, Boonstra FN, Riemslag FC, Ruiter M, de Vries BB, den Hollander AI, Collin RW, Cremers FP (2014). Nonpenetrance of the most frequent autosomal recessive leber congenital amaurosis mutation in NMNAT1. JAMA Ophthalmol.

[36] Sasaki Y, Margolin Z, Borgo B, Havranek JJ, Milbrandt J (2015). Characterization of Leber congenital amaurosis-associated NMNAT1 mutants. J Biol Chem.

[37] Roepman R, Letteboer SJ, Arts HH, van Beersum SE, Lu X, Krieger E, Ferreira PA, Cremers FP (2005). Interaction of nephrocystin-4 and RPGRIP1 is disrupted by nephronophthisis or Leber congenital amaurosis-associated mutations. Proc Natl Acad Sci USA.

[38] Rivolta C, Peck NE, Fulton AB, Fishman GA, Berson EL, Dryja TP (2001). Novel frameshift mutations in CRX associated with Leber congenital amaurosis. Hum Mutat.

[39] Weisschuh N, Feldhaus B, Khan MI, Cremers F, Kohl S, Wissinger B, Zobor D (2018). Molecular and clinical analysis of 27 German patients with Leber congenital amaurosis. PLoS ONE.

[40] Xu K, Xie Y, Sun T, Zhang X, Chen C, Li Y (2020). Genetic and clinical findings in a Chinese cohort with Leber congenital amaurosis and early onset severe retinal dystrophy. Br J Ophthalmol.

[41] Apte RS (2018). Gene therapy for retinal degeneration. Cell.

[42] Wang X, Yu C, Tzekov RT, Zhu Y, Li W (2020). The effect of human gene therapy for RPE65-associated Leber's congenital amaurosis on visual function: a systematic review and meta-analysis. Orphanet J Rare Dis.

[43] Cideciyan AV, Jacobson SG, Beltran WA, Sumaroka A, Swider M, Iwabe S, Roman AJ, Olivares MB, Schwartz SB, Komaromy AM (2013). Human retinal gene therapy for Leber congenital amaurosis shows advancing retinal degeneration despite enduring visual improvement. Proc Natl Acad Sci USA.

[44] Cideciyan AV, Jacobson SG, Drack AV, Ho AC, Charng J, Garafalo AV, Roman AJ, Sumaroka A, Han IC, Hochstedler MD, Pfeifer WL, Sohn EH, Taiel M, Schwartz MR, Biasutto P, Wit W, Cheetham ME, Adamson P, Rodman DM, Platenburg G, Tome MD, Balikova I, Nerinckx F, Zaeytijd J, Van Cauwenbergh C, Leroy BP, Russell SR (2019). Effect of an intravitreal antisense oligonucleotide on vision in Leber congenital amaurosis due to a photoreceptor cilium defect. Nat Med.

[45] Maeder ML, Stefanidakis M, Wilson CJ, Baral R, Barrera LA, Bounoutas GS, Bumcrot D, Chao H, Ciulla DM, DaSilva JA, Dass A, Dhanapal V, Fennell TJ, Friedland AE, Giannoukos G, Gloskowski SW, Glucksmann A, Gotta GM, Jayaram H, Haskett SJ, Hopkins B, Horng JE, Joshi S, Marco E, Mepani R, Reyon D, Ta T, Tabbaa DG, Samuelsson SJ, Shen S, Skor MN, Stetkiewicz P, Wang T, Yudkoff C, Myer VE, Albright CF, Jiang H (2019). Development of a gene-editing approach to restore vision loss in Leber congenital amaurosis type 10. Nat Med.

[46] Greenwald SH, Brown EE, Scandura MJ, Hennessey E, Farmer R, Pawlyk BS, Xiao R, Vandenberghe LH, Pierce EA (2020). Gene therapy preserves retinal structure and function in a mouse model of *NMNAT1*-associated retinal degeneration. Mol Ther Methods Clin Dev.

[47] Pellissier LP, Quinn PM, Alves CH, Vos RM, Klooster J, Flannery JG, Heimel JA, Wijnholds J (2015). Gene therapy into photoreceptors and Müller glial cells restores retinal structure and function in CRB1 retinitis pigmentosa mouse models. Hum Mol Genet.

